# Evolutionary Perspectives on Germline-Restricted Chromosomes in Flies (Diptera)

**DOI:** 10.1093/gbe/evab072

**Published:** 2021-04-23

**Authors:** Christina N Hodson, Laura Ross

**Affiliations:** Institute of Evolutionary Biology, University of Edinburgh, United Kingdom

**Keywords:** reproduction, non-Mendelian inheritance, chromosome elimination, programmed DNA elimination, accessory chromosome

## Abstract

In some eukaryotes, germline soma differentiation involves elimination of parts of the genome from somatic cells. The portions of the genome restricted to the germline often contain genes that play a role in development and function of the germline. Lineages with germline-restricted DNA are taxonomically diverse, and the size of the germline-restricted genome varies substantially. Unfortunately, few of these lineages have been studied in detail. As a result, we understand little about the general evolutionary forces that drive the origin and maintenance of germline-restricted DNA. One of the taxonomic groups where germline-restricted DNA has been poorly studied are the flies (Diptera). In three Dipteran families, Chironomidae, Cecidomyiidae, and Sciaridae, entire chromosomes are eliminated from somatic cells early in embryonic development. Germline-restricted chromosomes are thought to have evolved independently in the Dipteran families and their size, number, and transmission patterns vary between families. Although there is a wealth of cytological studies on these chromosomes in flies, almost no genomic studies have been undertaken. As a result, very little is known about how and why they evolved and what genes they encode. This review summarizes the literature on germline-restricted chromosomes in Diptera, discusses hypotheses for their origin and function, and compares germline-restricted DNA in Diptera to other eukaryotes. Finally, we discuss why Dipteran lineages represent a promising system for the study of germline-restricted chromosomes and propose future avenues of research on this topic.

**Significance**

Some animal species carry germline-restricted chromosomes, which are present in germ cells but eliminated from somatic cells early in development. Why and how these chromosomes evolve are unresolved questions in evolutionary biology. Three Dipteran families have germline-restricted chromosomes: nonbiting midges (Chironomidae), gall gnats (Cecidomyiidae), and black-winged fungus gnats (Sciaridae). Although there is over a century of cytological work on these chromosomes in Diptera, we know little about the genetics they encode. We review similarities and differences between germline-restricted chromosomes in the three Dipteran clades that contain them, discuss evolutionary theories on the origins of these chromosomes, and suggest some possible directions for future research on the evolution of germline-restricted chromosomes in Diptera.

## Introduction

Differentiation between the soma and the germline is a ubiquitous feature of multicellular animals. Germ/soma differentiation takes place very early in development and lays the groundwork for differences in gene expression and ultimately function between these two cell types (Beams and Kessel 1974). Although in most species, differences between the germline and the soma are of a regulatory nature, there are some lineages in which genetic as well as regulatory changes are associated with germline/soma distinction. These lineages have germline-specific DNA, which is eliminated from the soma (Wang and Davis 2014). Germline-specific DNA has evolved independently across the eukaryotic tree of life and has persisted in several large clades over extended evolutionary time (White 1973; Wang and Davis 2014; Torgasheva et al. 2019). Historically, germline-restricted DNA has been identified either because researchers observed the elimination of DNA from early somatic cells (Boveri 1887; Kahle 1908; Beermann 1959), additional chromosomes only present in the germline were detected (Nakai et al. 1991; Pigozzi and Solari 1998), or more recently, genomic data identified sequences found only in the germ cells (Smith et al. 2009). Given that detailed cytological studies have taken place in relatively few lineages, and that few studies specifically sequence germ tissue, it is possible that germline-restricted DNA is present in more eukaryotes and we have not detected it yet. Although germline-specific DNA seems to be functionally important, why germline-restricted DNA exists, how it evolves, and why it is present in some lineages but not others are unresolved questions in evolutionary biology.

Programmed DNA elimination from somatic cells produces germline-restricted DNA. This can happen in two ways, via chromatin diminution and/or chromosome elimination. Under chromatin diminution, which occurs in lampreys (Smith et al. 2009), hagfish (Nakai et al. 1991), nematodes (Boveri 1887), ciliates (Prescott et al. 1973), and copepods (Beermann 1959), specific breakpoints in the genome govern the loss of chromosomal regions. However, under chromosome elimination, which occurs in passerine birds (Pigozzi and Solari 1998), hagfish (Nakai et al. 1991), and flies (Kahle 1908), whole chromosomes are lost during early mitotic divisions in the embryo (Wang and Davis 2014). In both systems, germline-restricted DNA is lost from somatic cells early in development, but the mechanism of DNA elimination seems to be different for these two systems. The selection pressures driving both the initial evolution and the maintenance of germline-restricted DNA are not well established; and given the mechanistic differences between chromatin diminution and chromosome elimination, it is unclear whether the same forces lead to their origin. There are many ideas about how programmed DNA elimination initially evolves. Some ideas suggest that germline-restricted DNA evolves as a means of germline specialization, or to prevent expression of genes which would be potentially harmful if expressed in somatic cells, whereas others suggest that genomic conflict and the action of selfish genetic elements initially drives the evolution of germline-restricted DNA (Wang and Davis 2014; Smith, 2018; Hansson 2019). However, once portions of the genome are restricted to the germline, similar selection pressures likely act in chromatin diminution and chromosome elimination systems to restrict portions of the genome to the germline which are either beneficial in the germline or harmful in somatic cells (Bryant et al. 2016; Kinsella et al. 2019; Wang et al. 2020). Recent studies have developed methods to sequence and characterize regions of the genome restricted to the germline, by sequencing germ and somatic tissue and comparing the sequence composition of the two tissue types. These techniques have allowed us to learn more about the evolutionary history of germline-restricted DNA. Genomic studies have mostly been focused on groups with chromatin diminution (e.g., Chen et al. 2014; Sun et al. 2014; Smith et al. 2018; Wang et al. 2020), and studies on chromosome elimination have largely been confined to birds (Biederman et al. 2018; Kinsella et al. 2019). Cases of chromosome elimination in insects remain poorly studied, despite their discovery more than a century ago (Kahle 1908). Here we focus on germline-restricted chromosomes (GRCs) in flies (Diptera), where somatic chromosome elimination has evolved repeatedly and is present in three families (White 1973) (Box 1).

**Fig. 1.**
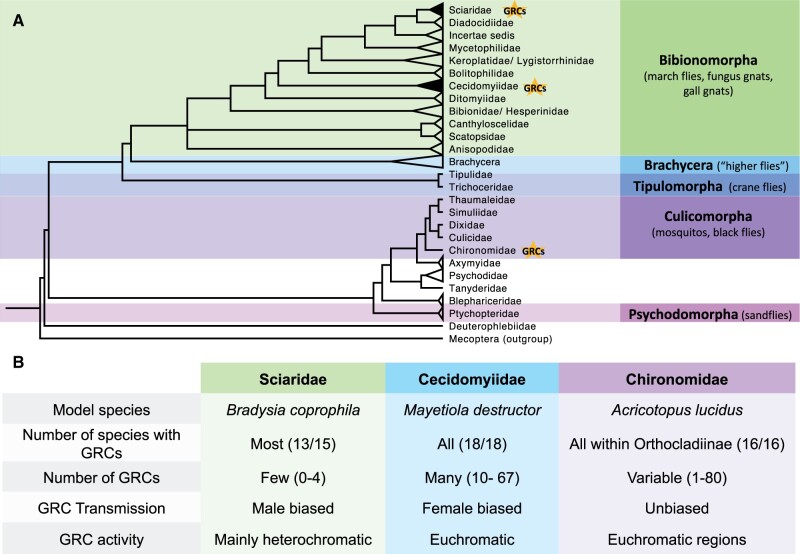
(*A*) Phylogeny of Dipteran families adapted from Ševčík et al. (2016) with permission, showing Dipteran families that carry GRCs (indicated with stars). Cecidomyiidae and Sciaridae are both part of the infraorder Bibionomorpha, whereas Chironomidae is a distantly related family in the infraorder Culicomorpha. Families in the suborder Brachycera are not shown for brevity. (*B*) A summary of GRC features in fungus gnats (Sciaridae), gall gnats (Cecidomyiidae), and nonbiting midges (Chironomidae).

Within flies, somatic chromosome elimination results in the presence of between 1 and more than 80 GRCs (table 1). GRCs have been identified in species within the Dipteran families Chironomidae, Cecidomyiidae, and Sciaridae (note: it is possible that other less well-studied Dipteran families also contain GRCs which have not been identified). Cecidomyiidae and Sciaridae are relatively closely related families of gall gnats and fungus gnats (both in the same infraorder Bibionomorpha), whereas Chironomidae is a more distantly related family of nonbiting midges (fig. 1). From their phylogenetic distribution, it appears that all three instances of GRCs have evolved independently (Ševčík et al. 2016). Although there is a long history of cytological studies on GRCs in Diptera (some of the earliest studies include: Kahle 1908; Metz 1925a; Bauer and Beermann 1952), studies are restricted to a small number of species and very little is known about the evolution of these intriguing chromosomes. For instance, it is still unclear how GRCs originated in the three different lineages, what their role might be, and whether these chromosomes are selfish, adaptive, or both. Additionally, although recent genomic approaches have started to address these questions in other systems with germline-restricted DNA, genomic research into the GRCs of Diptera is in its infancy. Therefore, we feel that it is time to revisit these chromosomes in Diptera. We review the current state of knowledge on GRCs in the three Dipteran lineages, summarizing the wealth of cytogenetic analyses, integrating this with the emerging genomic research in this field, comparing findings to other taxa with germline-restricted DNA, and discuss future directions and key questions remaining.

**Table 1:** Species in Sciaridae, Cecidomyiidae, and Chironomidae that Have Been Cytologically Examined for GRCs. For each species, we have included information about the system of chromosome inheritance (i.e., Mendelian vs. paternal genome elimination) and whether females of that species are monogenic (produce offspring of only one sex) or digenic (produce offspring of both sexes).

Family	Genus	Species	GRC	GRC No.	Somatic Karyotype	Inheritance	Sex Determination	Female Reproduction	References
Sciaridae	*Bradysia* (*Sciara*)	*coprophila*	Yes	2–3	2*n* = 8	PGE	XO	Monogenic	Metz (1938)
		*impatiens*	Yes	2–3	2*n* = 8	PGE	XO	Monogenic	Metz (1938)
		*varians*	Yes		2*n* = 8	PGE	XO	Monogenic	Metz (1938)
		*prolifica*	Yes	1–2	2*n* = 8	PGE	XO	Digenic	Metz (1938)
		*fenestralis*	Yes	1–2	2*n* = 8	PGE	XO	Mixed	McCarthy (1945)
		*ocellaris*	No	0	2*n* = 8	PGE	XO	Digenic	Metz (1938)
		*reynoldsi*	No	0	2*n* = 8	PGE	XO	Unknown	Metz (1938)
	*Corynoptera* (*Sciara*)	*subtrivialis*	Yes		2*n* = 8	PGE	XO	Monogenic	Metz (1938)
	*Lycoriella* (*Sciara*)	*similans*	Yes	2	2*n* = 8	PGE	XO	Mixed	Metz (1938)
		*mali* (*pauciseta*)	Yes	2	2*n* = 8	PGE	XO	Digenic	Metz (1938)
		*agraria*	Yes	2	2*n* = 8	PGE	XO	Digenic	McCarthy (1945)
	*Plastosciara*	*pectiventri*	Yes	1–4	2*n* = 8	PGE	XO	Unknown	Fahmy (1949b)
	*Rhynchosciara*	*hollaenderi*	Yes	1	2*n* = 8	PGE	XO	Monogenic	Mattingly and Dumont (1971)
	*Scatopsciara* (*Sciara*)	*nacta*	Yes	2	2*n* = 8	PGE	XO	Unknown	McCarthy (1945)
	*Trichosia*	*pubescens*	Yes	3–4	2*n* = 8	PGE	XO	Unknown	Amabis (1979)
Cecidomyiidae	*Aphidoletes*	*aphidimyza*	Yes	10–23	2*n* = 8	PGE	Unknown	Unknown	Gruzova and Batalova (1993)
	*Asphondylia*	*monacha*	Yes	∼50	2n = 8	PGE	Unknown	Unknown	White (1950)
	*Heteropeza*	*pygmaea*	Yes	48–67	2*n* = 10	PGE	Paedogenetic	Unknown	Panelius (1968), Kunz and Eckhardt (1974)
	*Lasioptera*	*rubi*	Yes	∼32	2*n* = 8	PGE	XXOO	Unknown	White (1950)
	*Mayetiola*	*destructor*	Yes	20–35	2*n* = 8	PGE	XXOO	Mixed	Stuart and Hatchett (1988)
	*Miastor*	*metrolas'*	Yes	36	2*n* = 12	PGE	Paedogenetic	Unknown	White (1946)
		sp.	Yes	36	2*n* = 12	PGE	Paedogenetic	Unknown	White (1946)
	*Mikiola*	*fagi*	Yes	∼16	2*n* = 8	PGE	Unknown	Unknown	Matuszewski (1961)
	*Monarthropalpus*	*buxi*	Yes	∼24	2*n* = 8	PGE	XXOO	Unknown	White (1950, 1973)
	*Mycophila*	*speyeri*	Yes	20–27	2*n* = 6	PGE	Paedogenetic	Unknown	Nicklas (1960), Camenzind (1971
	*Oligotrophus*	*pattersoni*	Yes	26	2*n* = 8	PGE	XXOO	Unknown	White (1950)
		*schmidti*	Yes	?	Unknown	Unknown	Unknown	Unknown	Matuszewski (1966)
	*Rabdophaga*	*saliciperda*	Yes	∼38	2*n* = 8	PGE	XXOO	Monogenic	Kraczkiewicz (1966)
		*salicisbatatas* (*batatas*)	Yes	30–32	2*n* = 8	PGE	XXOO	Monogenic	Geyer-Duszynska (1961)
	*Rhopalomyia*	*sabinae*	Yes	∼17	2*n* = 8	PGE	XXOO	Unknown	White (1950)
	*Taxomyia*	*taxi*	Yes	32	2*n* = 8	PGE	XXOO	Unknown	White (1947, 1973)
	*Trishormomyia*	*helianthi*	Yes	16	2*n* = 8	PGE	XXOO	Unknown	White (1973)
	*Wachtliella*	*persicariae*	Yes	∼32	2*n* = 8	PGE	XXOO	Unknown	Kunz et al. (1970)
Chironomidae	*Acricotopus*	*lucidus*	Yes	4–19	2*n* = 6	Mendelian	Unknown	Digenic	Staiber (2004)
	*Cardiocladius*	sp.	Yes	Up to 80	2*n* = 4	Mendelian	Unknown	Digenic	Beermann (1956)
	*Clunio*	*marinus*	Yes	∼8	2*n* = 6	Mendelian	Unknown	Digenic	Bauer and Beermann (1952)
	*Cricotopus* (*Eucricotopus*)	*ornatus* (*atritarsis*)	Yes	∼9	2*n* = 6	Mendelian	Unknown	Digenic	Bauer and Beermann (1952)
		*silvestris*	Yes	10–12	2*n* = 6	Mendelian	Unknown	Digenic	Bauer and Beermann (1952)
	*Halocladius (Trichocladius)*	*vitripennis*	Yes	5–7	2*n* = 6	Mendelian	Unknown	Digenic	Bauer and Beermann (1952)
	*Limnophyes*	sp.	Yes	∼8	2*n* = 6	Mendelian	Unknown	Digenic	Bauer and Beermann (1952)
	*Metriocnemus*	*martinii* (*cavicola*)	Yes	12–26	2*n* = 4	Mendelian	Unknown	Digenic	Bauer and Beermann (1952)
		*hygropetricus*	Yes	1–4	2*n* = 6	Mendelian	Unknown	Digenic	Bauer and Beermann (1952)
		*inopinatus*	Yes	6–8	2*n* = 4	Mendelian	Unknown	Digenic	Bauer and Beermann (1952)
		sp.	Yes	2–3	2*n* = 6	Mendelian	Unknown	Digenic	Bauer and Beermann (1952)
	*Psectrocladius*	*obvius*	Yes	1–4	2*n* = 6	Mendelian	Unknown	Digenic	Bauer and Beermann (1952)
		*platypus*	Yes	3–5	2*n* = 6	Mendelian	Unknown	Digenic	Bauer and Beermann (1952)
		*sordidellus* (*remotus*)	Yes	10–14	2*n* = 6	Mendelian	Unknown	Digenic	Bauer and Beermann (1952)
		sp.	Yes	∼6	2*n* = 6	1Mendelian	Unknown	Digenic	Bauer and Beermann (1952)
	*Smittia*	*parthenogenetica*	Yes	Up to 30	Unknown	Mendelian	Unknown	Digenic	Bauer (1970)
Culicidae	*Chaoborua*, *Culex*	2 species	No						Makino (1950)
Drosophilidae	*Drosophila*, *Scaptomyza*	23 species	No						Makino (1950)
Mycetophylidae	*Apolipthisa*, *Brachypeza*, *Exechia*, *Mycetophila*, *Rhymosia*	12 species	No						Makino (1950), Fahmy (1949a), Le Calvez (1947)
Sarcophagidae	*Sarcophaga*, *Tuberosa*	3 species	No						Makino (1950)
Syrphidae	*Eristalis*	1 species	No						Makino (1950)
Tachinidae	*Phorocera*	1 species	No						Makino (1950)

Note.—Species names from reference publication (when different from the current name) are in parentheses.

## Characteristics of GRCs in Diptera

Below, we outline some important features of GRCs in the three Dipteran families they occur in. GRCs are known by different names in each Dipteran lineage. To avoid confusion, and facilitate comparison between the different lineages, we refer to them as GRCs in all lineages after they are introduced. We will refer to chromosomes present in the germline and the soma—including both autosomes and sex chromosomes—as “core chromosomes.”

### Chironomidae

**Fig. 2:**
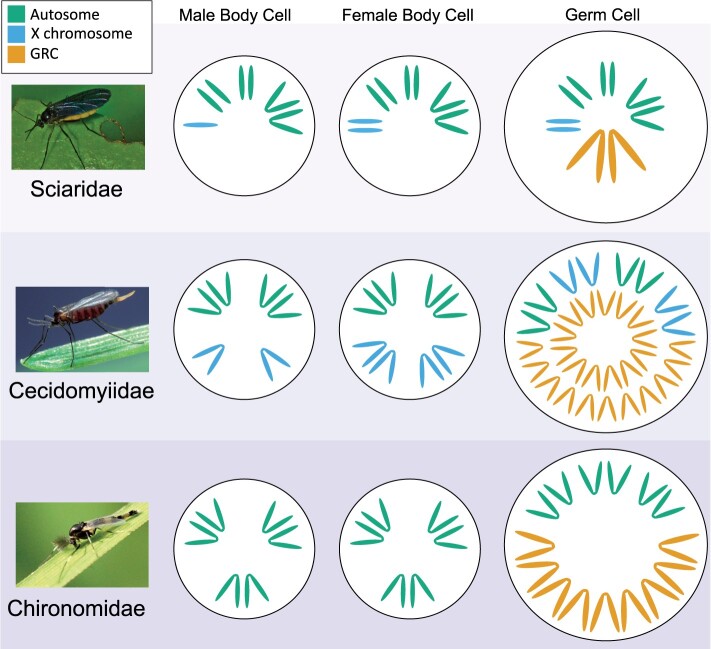
Differences in chromosome constitution between germline and somatic cells in the three Dipteran families with GRCs and representative images of members of these three families. Chromosome numbers are representative of the most well-studied member of each family: *Bradysia coprophila* for Sciaridae, *Mayetiola destructor* for Cecidomyiidae, and *Acricotopus lucidus* for Chironomidae. The chromosome numbers for other members of these families may be slightly different. The sex chromosome system in *A. lucidus* is not known, although mostly likely it has an unidentified homomorphic XY pair. Image of Chironomidae species (*Cricotopus trifasciatus*) attributed to James K. Lindsey and Cecidomyiidae species (*M. destructor*) attributed to Scott Bauer.

**Fig. 3:**
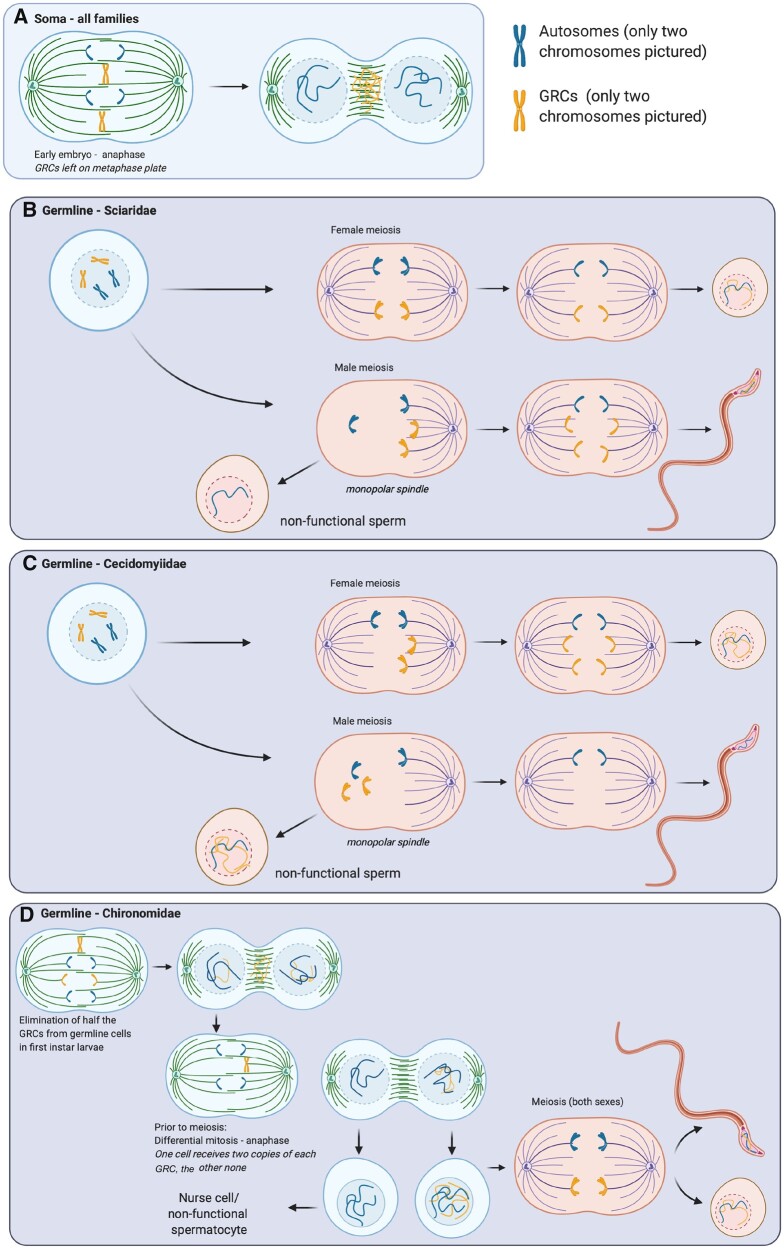
Behavior and transmission of the GRCs in each Dipteran lineage. (*A*) Somatic elimination of GRCs during embryogenesis (for all three lineages) where GRCs are left on the metaphase plate in early mitotic divisions. GRC transmission patterns during meiosis in (*B*) Sciaridae, (*C*) Cecidomyiidae, and (*D*) Chironomidae. In Sciaridae, GRCs exhibit male-biased transmission, in Cecidomyiidae, GRCs are maternally transmitted, and in Chironomidae, GRC transmission is unbiased but GRCs undergo an unusual elimination in early germ cells. Figure created with BioRender.com.

*Chironomidae* is a large family (∼10,000 species) of nonbiting midges with a global distribution (Armitage et al. 1995). It appears that GRCs are restricted to the subfamily Orthocladiinae, with 16 species within this subfamily found to carry GRCs (table 1, Bauer and Beermann 1952). The GRCs in Chironomidae are known as *K* chromosomes (short for “Keimbahn,” germline in German) and range in number from 1 to up to 80. Most of what we know about GRCs in this clade come from just one species, *Acricotopus lucidus* (fig. 2). In this species, there is a variable number of GRCs (*n* = 6–16), which are eliminated through lagging during early cleavage divisions whereas the core chromosomes segregate normally into daughter nuclei (Staiber 2004, 2008). Subsequently, in germline development, in a complex series of cell divisions, half of the GRCs are eliminated, but then the number is restored again in an unequal division just prior to meiosis (fig. 3, Staiber 2008). This process occurs in both sexes. The loss of half the GRCs is peculiar, and it is unclear why it occurs, if it is a random process or targeted at particular chromosomes. However, experiments tracking the inheritance of X-ray induced markers on the GRCs suggest it is not parent-of-origin specific (Staiber 1991; Staiber W, personal communication). Meiosis and gametogenesis occur as normal so that both sperm and eggs contain a haploid set of both the core chromosomes and the GRCs. The GRCs are more numerous than the core chromosomes (*n* = 3) and also appear larger (Staiber and Schiffkowski 2000). Detailed G-banding analyses in *A. lucidus* suggests that there are *n* = 9 distinct GRCs that occur in various frequencies and combinations within individuals (Staiber 1991). Some individuals only carry some of these nine chromosomes, whereas individuals with large numbers of GRCs have multiple copies of several of the GRCs (GRC polysomy). The GRCs pair during meiosis (either as bivalents or multivalents) and seem to recombine, but usually only within, not between the nine GRC chromosome types (Staiber 1989). GRCs show similarities in banding patterns with the core chromosomes and share homologous sections (Staiber and Schiffkowski 2000). This suggests that they are derived from the core chromosomes possibly through polyploidization. GRCs also occur in at least one parthenogenetic species, *Smittia parthenogenetica* (Bauer 1970).

### Cecidomyiidae

Cecidomyiidae is a family of gall gnats, with more than 6,000 species and a global distribution (Gagné 2010). Most species in this family are phytophagous (Stuart et al. 2008; Tokuda 2012). The most well-studied species cytologically is the Hessian fly *Mayetiola destructor* (fig. 2), which is a major pest on wheat (Stuart et al. 2008). GRCs were identified in Cecidomyiidae in 1908, when during early embryogenesis, the elimination of a large number of chromosomes from future somatic cells, but not germ cells was observed by Kahle in the midge *Miastor metrolas.* The GRCs in Cecidomyiidae are known as E chromosomes because they are “eliminated” from somatic cells. All Cecidomyiidae species investigated have been found to carry GRCs, with these chromosomes having been identified in 14 species from 13 Cecidomyiidae genera (table 1). Like in Chironomidae, GRCs in this lineage are numerous, ranging in number from 10 to more than 67 (table 1). The number of GRCs in Cecidomyiidae can also differ within species, suggesting that at least some of these chromosomes are accessory in germ cells.

GRCs in Cecidomyiidae are lost from somatic cells early in embryogenesis by being left on the metaphase plate at the fifth cleavage division (Geyer-Duszynska 1959; White 1973). GRCs are retained in the germ tissue of both sexes, but are only transmitted through eggs, except in one species, *Monarthropalpus buxi*, in which occasional GRC transmission through the sperm has been noted (White 1973). This is similar to the GRC in zebra finches, which is also maternally transmitted with rare occurrences of paternal transmission (Pigozzi and Solari 2005; Pei et al. 2021) Most Cecidomyiids with GRCs have an X_1_X_2_OO sex chromosome system (i.e., males have two distinct X chromosomes and females have two homologous pairs of X chromosomes). However, a few Cecidomyiid species with GRCs have paedogenetic reproduction instead, where females are diploid, males are haploid and there is an alternation between sexual and paedogenetic cycles. Paedogenetic females do not develop into adults, but instead go through an interesting development process where a females’ larvae develop within her body and eventually kills her. GRC behavior is similar in paedogenetic females and sexual females, so we discuss just sexual females below (White 1946). In meiosis in sexual females, GRCs often undergo a peculiar division different from the core chromosomes, where the GRCs form univalents and divide once (corresponding to the second division of the core chromosomes) (Matuszewski 1961; Stuart and Hatchett 1988). This causes the egg to have a full complement of GRCs, but also means that there is likely no recombination for GRCs in Cecidomyiidae species. In male meiosis, the GRCs are only present in the first division of meiosis and segregate with the paternally derived chromosomes into a bud which does not form into viable sperm (White 1946, 1947, 1973). This unusual GRC segregation in males is likely related to the unconventional reproduction system found in Cecidomyiidae, paternal genome elimination, where only maternally derived chromosomes are transmitted through males to future generations (see Reproduction and GRCs for more details) (Gallun and Hatchett 1969).

### Sciaridae

Sciaridae is a family of dark-winged fungus gnats, which live in moist environments and are familiar to many as small black gnats associated with houseplants (Mohrig et al. 2013). These insects have a global distribution with over 2,000 species (Mohrig and Menzel 2009). Sciaridae species, especially *Sciara coprophila* (now known as *Bradysia coprophila*) has been a focus of research since the 1920s, with particular interest in their unusual genetic system and giant polytene chromosomes (Metz 1925b; Gerbi 1986).

GRCs in Sciaridae were first discovered in 1925 by Metz in *B. coprophila*, when additional chromosomes were identified in male germ tissue (Metz 1925a). There was initially substantial confusion about GRCs in Sciaridae, as they were not identified in females and so thought to be sex chromosomes (Metz 1925a; Metz and Moses 1926). However, this misinterpretation was soon corrected upon GRCs being identified in female germ cells as well as male germ cells (Metz and Schmuck 1931). The GRCs in this clade are known as L chromosomes because they are germline “limited.” The most striking difference between GRCs in Sciaridae and Cecidomyiidae is that although Cecidomyiids have numerous GRCs, Sciaridae species have few, large GRCs, ranging in number from 1 to 4 (table 1). GRCs are thought to have evolved once in this lineage (Gerbi 1986; Haig 1993). Some sciarid species have also lost GRCs entirely, showing that these chromosomes are not necessary in some species within this lineage (Berry 1941; Gerbi 1986). GRCs have been identified in 13 species from 7 genera within Sciaridae and have been lost in two closely related species in the genus *Bradysia* (table 1). It is difficult to say how many times GRC loss has occurred in this clade as few species have been systematically studied for their presence.

Like in both Cecidomyiidae and Chironomidae, GRCs are eliminated from somatic cells early in development (5–6 cleavage division) by being left on the metaphase plate in mitosis and not incorporated in daughter nuclei (Du 1933). In germ cells, most of what we know about GRC behavior comes from *B. coprophila.* In this species, which initially has three GRCs in the zygote, one of these three chromosomes is lost in a mysterious manner early in germline development by seemingly being ejected from germ cells (Rieffel and Crouse 1966). Even in the somewhat rare case where the zygote initially has a different number of GRCs than three (presumably due to nondisjunction of GRCs in germ cell division prior to spermatogenesis), all but two of these chromosomes are eliminated from early germ cells (Rieffel and Crouse 1966). Transmission of GRCs in *B. coprophila* is generally male biased. In female meiosis, the two germline GRCs appear to pair normally, resulting in eggs that carry one GRC at the end of meiosis (Rieffel and Crouse 1966). However, Sciaridae has a similar type of reproduction as Cecidomyiidae, where meiosis is unconventional, and males only transmit maternally inherited chromosomes to offspring (Metz 1938). In Sciaridae, GRCs are not eliminated along with paternal chromosomes in the first division of meiosis like in Cecidomyiidae, instead these chromosomes segregate with the maternal chromosomes and all GRCs (normally two) are incorporated into viable sperm. There is some variation in the number of GRCs in sperm, ranging from 0 to 4, with the majority (78%) of sperm containing two GRCs (Rieffel and Crouse 1966). However, differences in GRC number are corrected in the next generation when all but two GRCs are eliminated from germ cells (Rieffel and Crouse 1966).

## Reproduction and GRCs

GRCs often have different modes of inheritance from the core chromosomes in the genome. Because of this, understanding how chromosome transmission and reproduction occurs in species with GRCs helps to establish the mechanism by which GRCs persist. In Dipterans, this is particularly interesting because two of the three Dipteran lineages with GRCs have unusual, but very similar reproduction systems.

### Non-Mendelian Inheritance and GRCs

Both Sciaridae and Cecidomyiidae have independently evolved unconventional reproduction systems, which bear a striking resemblance to each other (Anderson et al. 2020) (fig. 3). Both families exhibit a form of asymmetric chromosome inheritance known as paternal genome elimination (Metz 1938; Gallun and Hatchett 1969; Gardner and Ross 2014; Burt and Trivers 2006). In paternal genome elimination, although males develop from fertilized eggs, they exclusively transmit maternally derived chromosomes to their offspring. Females transmit chromosomes in a typical Mendelian manner (with the possible exception of the GRCs). In contrast to Sciaridae and Cecidomyiidae, core chromosomes in Chironomidae follow classic Mendelian transmission, although inheritance patterns of the GRCs are less clear: Around half of all GRCs are eliminated from early germline cells and it is possible that this elimination might be nonrandom (Staiber 1991). It is currently unclear if and how paternal genome elimination is related to the evolution of GRCs, although it seems plausible that they may be related, as the only two fly lineages with paternal genome elimination also carry GRCs.

Both Sciaridae and Cecidomyiidae also have an unusual type of sex determination in which X chromosome elimination early in development governs whether an individual is a male or a female (Du 1933; White, 1973). Sciaridae has an XO sex chromosome system, whereas Cecidomyiidae has an X_1_X_2_OO system, but interestingly, in both lineages, the genotype of the mother seems to be the factor that controls offspring sex (Metz and Schmuck 1929; Benatti et al. 2010). In Sciaridae, this fact was established in *B. coprophila*, whereas in Cecidomyiidae, it was established in *M. destructor*. *Bradysia coprophila* has an inversion on the X chromosome which is only ever found in females (Crouse 1977). Females with the inversion produce only female offspring (genotype XX′, where X′ is the X chromosome with the inversion), whereas females without the inversion (genotype XX) produce only male offspring (with males having an XO genotype) (Gerbi 1986). Many other Sciaridae species have a similar system where females only produce offspring of one sex (i.e., females are monogenic), and these species are assumed to have a similar sex determination mechanism. However, other species have females that produce offspring of both sexes (i.e., females are digenic), or a mix of monogenic and digenic females, and the mechanism of sex determination in digenic species is less clear (Nigro et al. 2007). Similarly, In Cecidomyiidae, females can either be female producing, male producing, or digenic, but studies on *M. destructor* showed that the inversion that governs female production is on an autosome rather than the X chromosome (Benatti et al. 2010).

Anecdotally, monogeny seems to be associated with the presence of GRCs in Sciaridae. For instance, the most well-studied species without GRCs in Sciaridae, *Bradysia ocellaris*, has a digenic reproduction system, and many species with GRCs have monogenic reproduction (Gerbi 1986). Additionally, a monogenic lab line of *B. impatiens* which lost its GRCs through artificial selection, first became digenic, and later died out due to exclusive male production (Crouse et al. 1971). It is tempting, therefore, to speculate that the GRCs in Sciaridae are involved in some way in sex determination in species with monogenic reproduction. However, we do not have enough information about the taxonomic distribution of monogeny and GRCs to say much about whether these two factors are related and how and whether GRCs are involved in sex determination. In Cecidomyiidae, we also see a mix of species with exclusive monogeny, digeny, and a mix of both systems, but as species in this family always have GRCs, if GRCs are involved in sex determination, they are likely involved in a different way than in Sciaridae. Identifying whether genes on GRCs are involved in sex determination would provide valuable information about their importance in reproduction.

### Sex-Biased Transmission of GRCs

Identifying the way GRCs are transmitted (through males, females, or both) is not just important to understand how they evolved (see Origin and Evolution of GRC in Diptera section) but can also provide more general insight into the evolution of chromosomes with sex-biased transmission. The three families with GRCs each show a different pattern of GRC transmission. Cecidomyiidae species generally have strictly maternal transmission of the GRCs to offspring (White 1973), Sciaridae shows some variation, but in *B. coprophila*, two GRCs are transmitted through sperm, whereas only one is transmitted through the egg (which suggests male-biased transmission) (Rieffel and Crouse 1966). Finally, in Chironomidae, transmission appears unbiased with regard to sex. Because of the variability between clades (but also within clades), these chromosomes offer an opportunity to understand how sex-biased transmission affects the evolution of chromosomes which are not sex chromosomes. For instance, we would predict that the GRCs in *B. coprophila* might accumulate genes that benefit males, whereas the GRCs in Cecidomyiidae might accumulate genes that benefit females due to their different transmission patterns. Investigating whether this is actually the case, and whether these genes display other patterns that we expect from chromosomes with sex-biased transmission is an interesting avenue of further research.

GRC transmission is also important as it will affect if and how much recombination occurs between the GRCs. Since recombination only occurs during female meiosis in many Dipterans (including Cecidomyiidae and Sciaridae, but not Chironomidae; White 1973; Blackmon et al. 2017), GRCs in Cecidomyiidae and Sciaridae only have potential to recombine in females. In Cecidomyiidae, GRCs are maternally transmitted, however, GRCs segregate in an unusual manner in female meiosis, such that GRCs only form a univalent (as opposed to a bivalent where recombination may occur) (Stuart and Hatchett 1988). This suggests that GRCs in this clade should not recombine, although this idea remains to be tested with genomic data. In contrast, in Sciaridae, cytological evidence shows that GRCs generally form a bivalent during female meiosis (Crouse et al. 1971). Yet, recent genomic evidence suggests that there is likely very little recombination between the two GRCs in *B. coprophila* (Hodson et al. 2021). This could indicate that recombination of GRCs is restricted to a small portion of the chromosome (similar to highly diverged sex chromosomes) or may indicate instead that there is no recombination between these chromosomes despite the fact that they seem to form a bivalent during female meiosis. In Chironomidae, however, we would expect recombination to occur between the GRCs. In this clade, there is cytological evidence that some GRCs are homologous to each other and bivalents and chiasmata form during meiosis (Staiber 1991; Staiber and Wahl 2002). The level of recombination between GRCs is important to understand how they evolve over time. For instance, we would expect selection to be less efficient on GRCs that do not recombine and as a result for these chromosomes to perhaps accumulate transposable elementss and other repetitive elements.

## Function of GRCs in Diptera

The function of GRCs in Diptera remains largely unknown. In Sciaridae and Cecidomyiidae, there is evidence that GRCs are necessary in the species that contain them, but very little is known about their function besides this, and in Chironomidae, experimental studies on GRC function are lacking. In Cecidomyiidae, individuals that develop without GRCs have gonads which do not function normally (Geyer-Duszynska 1959; Bantock 1970). This is known from individuals that have been experimentally manipulated to lose their GRCs (however, it is also possible that the manipulation itself affected gonad function as suggested by Stuart and Hatchett 1988). Bantock (1970) conducted a series of experiments in *M. destructor* manipulating early embryos so that GRCs were eliminated from germ cells as well as somatic cells (through irradiation, centrifugation, and physical manipulation of embryos). Offspring that developed as a result of these manipulations appeared physically normal, but both males and females were unable to produce viable gametes. This suggests that the GRCs in Cecidomyiidae have a function relating to the production of gametes and are necessary for this process.

Similarly, in Sciaridae, we only know about the function of GRCs from what happens when they are experimentally eliminated from germ tissue. Early cytology studies on *Bradysia* species noted that the GRCs sometimes varied in size in different individuals and between species (Metz 1938). Crouse et al. (1971) used this fact to produce a line of *B. impatiens* with no GRCs. This line was only viable for a short time in the lab, as females with no GRCs lost the ability to produce monogenic (single sex) offspring and eventually produced exclusively male offspring. Additionally, Crouse et al. (1971) conducted reciprocal crosses between individuals carrying GRCs to individuals with no GRCs and found that some of these crosses resulted in incompatibilities, such that the offspring had mosaic gonads. Interestingly, *B. impatiens* is closely related to a sciarid species without GRCs, *B. ocellaris*. The authors suggest that as *B. impatiens* is closely related to a species without GRCs, it may be on an evolutionary trajectory toward GRCs becoming dispensable. Regardless, this study suggests that although GRCs are not strictly necessary for viability in *B. impatiens*, they seem to play a role in gonad maturation, gonad function, and potentially sex determination.

Genomic data from GRCs will aid future work addressing the function of these chromosomes. Once we identify genes on these chromosomes, we can use RNAseq data to explore expression patterns at different times in development, and genome manipulation strategies such as CRISPER-Cas9 and RNAi to knock out genes which may be functionally important. Gene knockout studies will allow us to pinpoint genes that are important for specific GRC behaviors (i.e., sex-biased transmission, elimination from somatic cells, etc.).

## Epigenetic Modifications and Chromatin Structure of the GRCs

In all three Dipteran families, the GRCs display unusual patterns of heterochromatization and epigenetic modifications. These modifications seem to be important for a number of key aspects of these chromosomes, namely GRC activity, GRC elimination from somatic cells, and GRC transmission and parent-of-origin effects.

### Heterochromatin and GRC Activity

GRCs in all three Dipteran families have a different appearance to the core chromosomes and show different patterns in the timing of replication. Cytological studies have focused on the level of condensation (heterochromatization) of GRCs as a proxy of whether they are likely active in cells. All three families show slightly different patterns of GRC heterochromatization, but it is not clear to what extent GRC chromatin level correlates with transcription levels of genes on these chromosomes, RNAseq data would be needed to determine this.

In the Cecidomyiid species *Taxomyia taxi* and *Miastor* sp., for instance, GRCs are generally diffuse (i.e., not heterochromatic) in male and female germ cells before meiosis takes place, but interestingly core chromosomes seem to be heterochromatic in this tissue (White 1946, 1947). This may indicate that GRCs are transcribed in germ cells, but that core chromosomes may not be. In the Chironomid *A. lucidus*, however, GRCs are more heterochromatic than core chromosomes. Staiber and Thudium (1986) note that GRCs have distinct euchromatic regions, and these regions stain for H3K4Me3, a histone modification associated with active chromatin, suggesting that the euchromatic regions likely contain expressed genes (Staiber 2012). In addition, it appears that core chromosomes are silenced during male meiosis whereas the GRCs are active. Finally, in Sciardae, GRCs are rich in heterochromatin and are nearly always heterochromatic, and therefore may not be transcriptionally active over much of development. The only time in which GRCs seem to be diffuse in *B. coprophila*, and therefore presumably the only times they are transcriptionally active, is in late embryo and early larval stages (which may reflect a role of these chromosomes in germ cell maturation) (Rieffel and Crouse 1966). Additionally, in another sciarid, *T. pubescens*, GRCs were found to also be diffuse in the period between meiosis I and meiosis II in males, which may indicate that they are also transcriptionally active at this time (Amabis et al. 1979).

### GRC Elimination and Histone Modifications

GRC elimination from somatic cells occurs in a strikingly similar manner in the three Dipteran lineages, by GRCs being left on the metaphase plate in early mitotic divisions (at the 5–6 cleavage division). Determining the underpinnings of this behavior will allow researchers to conclude whether this is a fascinating example of convergent evolution. Histone modifications appear to play a key role in GRC elimination from somatic cells. In *B. coprophila*, GRC (and X chromosome) elimination from somatic cells occurs through a failure of sister chromatids to separate in early mitotic divisions. de Saint Phalle and Sullivan (1998) noted that the centromeres appeared to be attached to spindles in mitosis, but chromatids were unable to separate from each other (and move to the daughter nuclei) as the chromosome arms seemed to be attached. It was later found that this separation failure corresponded to abnormalities in H3S10 phosphorylation (Escribá and Goday 2013). Generally, H3S10 becomes dephosphorylated in the metaphase to anaphase transition, which is associated with the separation of chromosomes in anaphase. However, in *B. coprophila*, the GRCs remain phosphorylated at H3S10, especially along the chromosome arms, which is associated with a failure of the chromosome arms to separate from each other and GRCs being eliminated because they are not incorporated into daughter nuclei. Interestingly, abnormalities in H3S10 dephosphorylation occur in a similar manner in chromatin eliminated from somatic cells in *Ascaris* nematodes, suggesting the mechanism of DNA elimination from somatic cells may be similar in these two lineages (Wang et al. 2020). In the Chironomid *A. lucidus*, GRCs elimination also occurs through sister chromatids not dividing in mitosis due to the chromosome arms not separating (Staiber 2006). However, in Chironomidae and Cecidomyiidae, less histone modification work has taken place during GRC elimination from somatic cells, so it is unknown whether exactly the same mechanism is involved in GRC elimination. One major benefit of studying GRCs in Diptera is that early embryogenesis (i.e., GRC elimination) is easy to observe (unlike other lineages with GRCs such as songbirds) and that the mechanism of chromosome elimination seems to be similar in three independent lineages. Therefore, future work on GRC elimination in Dipterans can provide information about whether similar mechanisms are involved in GRC elimination in different taxa.

### GRC Transmission and Parent of Origin Effects

The GRCs in Cecidomyiidae and Sciaridae display different transmission patterns, especially in male meiosis, where GRCs are not transmitted through the sperm in Cecidomyiidae but always transmitted through sperm in Sciaridae. Parent of origin effects may be important in these transmission differences. Both Sciaridae and Cecidomyiidae exhibit paternal genome elimination, where epigenetic markings often differ between chromosomes depending on whether they are inherited maternally or paternally, and these differences are likely important for chromosome segregation (Prantera and Bongiorni 2012). Therefore, retention or elimination of GRCs in male meiosis may be related to differences in epigenetic markings on the GRCs. There is conflicting evidence for this idea in the Sciarid *B. coprophila*, the only species for which data are available*.* In *B. coprophila*, like the paternally derived chromosomes, the GRCs are hypoacetylated on H3 and H4 in early meiosis in males (Goday and Ruiz 2002). However, both GRCs and maternally derived chromosomes stain densely for H3T11-P, whereas paternally derived chromosomes do not stain for this histone modification (Escribá et al. 2011). GRCs showing similar epigenetic markings to the maternally inherited chromosomes in male meiosis may indicate how they are able to segregate with the maternal chromosomes during meiosis. However, it is unclear at the moment how these differences play into GRC transmission and more research is clearly needed.

## Genomic Characterization of GRCs in Diptera

Genomic characterization of germline-restricted DNA has taken place in zebra finches (Biederman et al. 2018; Kinsella et al. 2019), nematodes (Wang et al. 2012, 2020), lampreys (Smith et al. 2018), copepods (McKinnon and Drouin 2013; Sun et al. 2014), and ciliates (Chen et al. 2014). Identifying germline-restricted DNA is often done by sequencing genomic DNA from germ tissue and somatic tissue separately and identifying regions of the genome that are at a higher coverage level and/or restricted to the germline sequence library, or regions that have short DNA sequences (either k-mers or single nucleotide polymorphisms) which are specific to the germline sequence library. This can be done with a genome assembly that is assembled with or without the germline-restricted sequence library. Few studies have attempted to sequence the germ tissue of Dipterans with GRCs. Although there are a handful of genome assemblies available for species within each family (five species in Chironomidae, four species in Cecidomyiidae, and one species in Sciaridae on NCBI as of October 22, 2020), as well as various gDNA and RNA data sets available for each group, nearly all of these data sets target the whole body of individuals, which contains predominantly somatic tissue. As a result, it is unlikely this data will be useful for GRC studies, as it likely contains a very small fraction of sequences from GRCs.

There are two studies, one in the Cecidomyiid *M. destructor* and one in the Sciarid *B. coprophila*, which attempt to sequence GRCs. Zhao et al. (2015) sequenced early embryos (before GRC elimination) in *M. destructor* and mapped reads back to the *M. destructor* reference genome. They found that overall mapping rates are similar between read libraries containing the GRCs and libraries that do not contain these chromosomes, and suggested that GRCs must therefore be composed of the same sequences as the core chromosomes. Further work, however, is needed to characterize the GRCs in this lineage as the experimental design and data quality were insufficient for more in-depth analyses. In the Sciarid *B. coprophila*, a recent study (Hodson et al. 2021) conducts an in-depth investigation of GRC content. This study identified sequences belonging to the GRCs and found that the GRCs in *B. coprophila* are large and gene-rich, containing ∼15,000 genes, many of which have paralogs on core chromosomes. This study found that GRCs have paralogs on all three autosomes and the X chromosome in roughly equal proportions, showing that there is no clear evidence for the GRCs having evolved from one specific core chromosome. This is similar to findings in zebra finches, where the GRC also seems to contain genes with paralogs distributed throughout the genome (Kinsella et al. 2019).

Future studies can use the established techniques for other species with germline-restricted DNA to sequence GRCs in Dipterans (especially Cecidomyiid and Chironomid species for which GRCs have not yet been characterized in detail). Collecting high coverage sequence data from tissue that contains GRCs (either from germ cells or embryos before GRC elimination) and comparing the genomic composition to tissue that does not contain GRCs is a robust method to identify germline-restricted sequences. This technique seems to work well even when the core chromosomes and GRCs share sequence similarity (Kinsella et al. 2019).

## Origin and Evolution of GRCs in Diptera

### Patterns and Theories of GRC Evolution in Diptera

Cytological observations on the presence of GRCs in Dipterans suggest that GRCs have evolved independently in Chironomidae, Cecidomyiidae, and Sciaridae (White 1973). This is very likely true for Chironomidae, given the phylogenetic distance between Chironomidae and Sciaridae/Cecidomyiidae (i.e., Chironomidae is in a different infraorder to Sciaridae/Cecidomyiidae) (fig. 1). Cecidomyiidae and Sciaridae are in the same infraorder Bibionomorpha, however, they are relatively divergent families within this infraorder (Ševčík et al. 2016). Although few other Bibionomorpha families have been examined in depth for the presence of GRCs, they are absent from Mycetophilidae, a family closely related to Sciaridae (branching between Sciaridae and Cecidomyiidae in phylogenies). In this family, 11 species in 5 genera have been examined and GRCs were absent in every species (Le Calvez 1947; Fahmy 1949a). Overall, this has led to the assumption that GRC evolution is independent in Sciaridae and Cecidomyiidae. It is currently unclear whether this is the case.

**Fig. 4:**
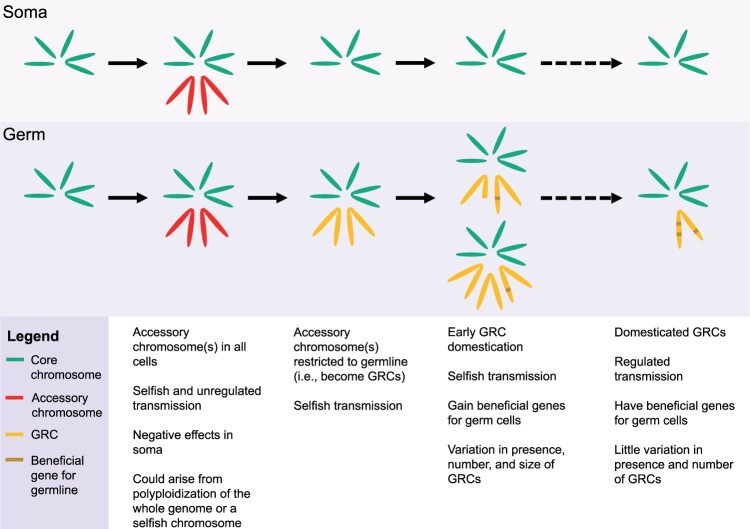
Possible trajectory of GRC evolution in germ and somatic cells. First, accessory chromosomes (those in addition to the core chromosomes) arise through some means (e.g., through whole-genome duplication or selfish chromosomes). These chromosomes are initially found in all cells but over time are restricted to the germline to mitigate negative effects of chromosome polyploidy in the soma (i.e., they become GRCs). The GRCs initially show variation, especially in number (due to non-Mendelian chromosome inheritance patterns). Over time, these chromosomes may gain beneficial genes for the germline and become “domesticated,” such that transmission and GRC number is regulated.

There are a number of theories for the initial evolution of GRCs in Dipterans and in other metazoa with GRCs. In Dipterans with many GRCs (i.e., Cecidomyiidae and Chironomidae), most theories focus on GRCs arising through whole-genome duplication (White 1949; 1973; Staiber and Schiffkowski, 2000); whereas in Sciaridae, which have fewer GRCs, theory suggests that GRCs originated from a selfish sex chromosome (Haig 1993). In other metazoans with GRCs, namely songbirds, GRCs are thought to have evolved from a supernumerary B chromosome (i.e., a nonessential chromosome in addition to the core chromosomes) (Hansson 2019). For all of these possible origins of GRCs, theory suggests that GRCs initially were present in all cells and shared similarity to the core chromosome set but were restricted to the germline to avoid possible negative effects of polysomy (fig. 4). The fact that in all three Dipteran families (as well as other metazoans with GRCs), GRCs are initially present in all cells but early in development (i.e., before zygote gene expression) they are eliminated from somatic cells seems to support this view. The non-Mendelian chromosome inheritance patterns of GRCs in both Sciaridae and Cecidomyiidae and the variation in GRC number in all three families has raised the suggestion that these chromosomes likely evolved as genomic parasites that may be in the process of being domesticated (fig. 4). Thus, the ongoing evolution of these chromosomes likely involves a balance between the interests of the host and the interests of the GRCs. Below, we summarize a few of the main ideas for initial GRC evolution in Diptera and outline factors supporting these ideas.

### GRC Evolution through Selfish Chromosomes

One possible origin of GRCs in Diptera is through selfish chromosomes. Selfish chromosomes exhibit non-Mendelian inheritance patterns and are transmitted to more than 50% of an individual’s offspring. Driving X chromosomes, for example, are found in many *Drosophila* species (as well as other Dipteran lineages) and cause the destruction of Y bearing sperm in males that carry them, causing the driving X chromosome to be transmitted through sperm more often than would be expected in Mendelian inheritance (Helleu et al. 2015). One of the most detailed ideas for how GRCs may have arisen in Sciaridae also involves a driving X chromosome (Haig 1993). This theory suggests that the evolution of GRCs and the non-Mendelian inheritance system in Sciaridae are related, and that the evolution of reproduction in Sciaridae involved intragenomic conflict between several genomic entities. Specifically, it suggests that a driving X chromosome (similar to those found in *Drosophila*) evolved in an ancestor of Sciaridae, and the maternally inherited chromosomes evolved to drive with the driving X chromosome, causing a paternal genome elimination-like system to evolve. This was followed by a shift in the sex determination system such that X chromosome elimination early in development governs the sex of an individual. This idea is supported by recent genomic evidence showing that the X chromosome in Sciaridae likely evolved at the base of this lineage (Anderson et al. 2020). Then GRCs evolved from paternally derived X chromosomes in males as a means to escape elimination during male meiosis (since paternally derived chromosomes in males are not transmitted to future generations under paternal genome elimination). The extra X chromosome(s) became restricted to the germline since X chromosomes polysomy in somatic cells may be detrimental. This theory contains some testable predictions about GRC evolution, namely that they are expected to have evolved after the divergence of Sciaridae from their sister clade (as this family does not exhibit paternal genome elimination) and that they evolved from the X chromosome and therefore would be expected to contain genes derived from this chromosome. However, a recent study found that GRCs in the Sciarid *B. coprophila* do not bear significant homology to the X chromosome in this species, and instead have homologous regions to all chromosomes in the core genomes, suggesting that in Sciaridae, at least, the GRCs do not seem to have evolved from the X chromosome (Hodson et al. 2021).

Another type of selfish chromosome which could give rise to GRCs are B chromosomes. B chromosomes are nonessential chromosomes present in some individuals within a species. These chromosomes are widespread in eukaryotes, present in 15% of species and found in at least 520 insect species (although they are not known to occur in Sciaridae, Cecidomyiidae, or Orthocladiinae) (Ahmad and Martins 2019). B chromosomes, like GRCs, can also show sex biased transmission, and can vary in number within individuals (Camacho 2000; Burt and Trivers 2006). These chromosomes are expected to arise through genomic conflict, by gaining a transmission advantage during meiosis despite potentially not carrying genes beneficial to the individuals that carry them. It is possible that GRCs arose from B chromosomes which became restricted to the germline. This idea is somewhat similar to Haig’s theory (1993) for the evolution of GRCs in Sciaridae but does not require the GRCs to evolve from any particular chromosome and has also been suggested as a possible origin of the GRC found in birds (Hansson 2019).

### GRC Evolution through Polyploidization

Alternately, GRCs in Diptera may have evolved through polyploidization. In both Chironomidae and Cecidomyiidae, GRCs are thought to have evolved through whole-genome duplication, followed by restriction of the extra chromosomes to the germline to mitigate negative effects of polyploidy in somatic cells. Although polyploidization is generally rare among Diptera (Román-Palacios 2020), there is evidence for polyploidization in Chironomidae species that do not carry GRCs, where there are several triploid parthenogenetic species (Carew 2013). In the chironomid *A. lucidus*, the fact that there are nine distinct GRCs that appear to have evolved from the three core chromosomes seem to support their origin through whole-genome duplication (White 1973; Staiber and Schiffkowski 2000). However, even if this is the case, there is clear evidence that they have since diverged from the core chromosome set as there are GRC-specific rearrangements and accumulation of repetitive DNA sequences located in pericentromeric and terminal heterochromatic segments that are not present in core chromosomes (Staiber and Schiffkowski 2000; Staiber 2017).

In Cecidomyiidae, as well, GRCs were originally thought to arise through whole-genome duplication (White 1946; Nicklas 1960). This idea arose from observations of GRC behavior in a number of Cecidomyiidae species. For instance, in both *Rhabdophaga saliciperda* and *Wachtliella persicariae*, it was observed that the GRC number was generally a multiple of the core chromosome number (*n* = 4) and additionally that GRCs spatially segregate into groups with four chromosomes each in meiosis, which was thought to indicate that chromosomes from each whole-genome duplication event segregate together during meiosis (Kraczkiewicz 1966; Kunz et al. 1970). This idea, however, is controversial in Cecidomyiidae, as GRCs generally do not look like core chromosomes. For instance, GRCs in *M. destructor* are all of different sizes and display different banding patterns, suggesting that GRCs are not homologous to core chromosomes or if they are, that they have diverged significantly (Stuart and Hatchett 1988). However, more recent genomic analyses fail to identify GRC-specific sequences in *M. destructor*, suggesting that the GRCs might have a similar genomic composition to the core chromosomes (Zhao et al. 2015). It is possible that both of these ideas are correct, and that GRCs in Cecidomyiidae originally evolved from the core chromosome set through whole-genome duplication but have undergone rearrangements and diverged over time so they no longer resemble the core chromosome set. More detailed genomic work, sequencing the GRCs at a higher coverage in *M. destructor*, would allow researchers to use similar techniques to those employed to characterize the GRCs in zebra finches (Kinsella et al. 2019), to disentangle whether the GRCs share homology to the core chromosome set, and whether portions of the core chromosome set, or entire chromosomes are similar to the GRCs.

Another possible mechanism that could have led to GRC evolution through polyploidization is hybridization. If the GRCs are of hybrid origin, their elimination from the soma might have evolved to reduce negative fitness consequences associated with hybrid incompatibilities. A recent study suggests that the GRCs in Sciaridae may have arisen due to hybridization between the ancestor of Sciaridae and a Cecidomyiid (Hodson et al. 2021). However, as Cecidomyiids also have GRCs, it is currently unclear whether the GRCs in this lineage, or the core chromosome set, introgressed into Sciaridae. B chromosomes in several lineages (e.g., *Nasonia* wasps, bees, etc.) have also been found to be of hybrid origin, suggesting that this may not be such an uncommon mechanism by which chromosomes with non-Mendelian inheritance can evolve (McAllister and Werren 1997; Tosta et al. 2014). How GRCs evolved in Sciaridae (i.e., whether they evolved from the Cecidomyiid core chromosomes or GRCs) is important for understanding whether there are one or two origins of GRCs in Diptera. As such, future work on this topic is needed.

Overall, it is far from clear how the GRCs arose in any of the Dipteran taxa that carry these chromosomes. Future genomic studies will help us to determine their origin. Specifically, knowing more about whether GRCs have paralogs on core chromosomes or other GRCs—within and between species, and also between different families with GRCs (specifically between Sciaridae and Cecidomyiidae)—will help resolve whether the origin of GRCs in Diptera are through whole-genome duplication, hybridization, or from selfish chromosomes. Once GRCs evolve, selection should favor their gaining a beneficial function for the germline over time, even if they originally evolved as a selfish chromosome such as a B chromosome or driving chromosome (fig. 4). Genomic studies, including gene expression and population genomic analyses, can help determine whether GRCs, and the genes they carry have gained a beneficial role in the species they occur in after they originated, as very little is known about the function of these chromosomes (see above).

## How Similar Are Dipteran GRCs to Metazoans with Germline-Restricted DNA?

Recent genomic work on lineages with germline-specific DNA has shown that germline-restricted DNA contains numerous protein-coding genes (Wang et al. 2012; Smith et al. 2018; Kinsella et al. 2019). Additionally, in some lineages, namely zebra finches, nematodes, and lampreys, germline-restricted genes are expressed and have functions relating to germline development and reproduction (Wang et al. 2012; Bryant et al. 2016; Kinsella et al. 2019). Recent work on the GRCs in Diptera, specifically *B. coprophila*, shows that these chromosomes carry a large number of protein-coding genes, although it remains to be seen whether these genes are expressed and have a similar function to germline-restricted genes in other lineages (Hodson et al., 2021).

The only lineage with GRCs which has been studied in depth are the passerine birds, specifically zebra finches, which have a large GRC that is maternally transmitted like in Cecidomyiidae, and which contains numerous genes that often function in female gonad development (Pigozzi and Solari 1998; Kinsella et al. 2019). In passerine birds, GRC evolution seems to have occurred in a slightly different way to Dipteran lineages. One large difference is that GRC evolution seems to have occurred once in songbirds, with all species having one GRC that may vary substantially in size and gene content between species, but seems to be present across the clade (Torgasheva et al. 2019). Therefore, GRC transmission and retention seem to be more regulated in songbirds than in Dipterans, which show greater variation in the number of GRCs in all families and also show some variation in the presence of GRCs. Songbirds and flies with GRCs offer two powerful systems to understand the evolution of GRCs, which can be used to tackle different questions about GRC evolution. The single origin of GRCs in songbirds and the retention of one GRC over time make this system better to answer questions about the ongoing evolution of a GRC chromosome, while the fact that there are several origins of GRCs in Diptera, and that there is variation in the presence, number, and size of GRCs, make this system potentially better for comparative studies of why GRCs evolve and are retained.

With continued efforts to characterize germline-restricted DNA in species with both chromatin diminution and chromosome elimination, we can begin to understand what aspects of these two systems are similar and what aspects are different. For instance, in species with chromatin diminution, repetitive DNA is often a large component of the somatically eliminated chromatin (Sun et al. 2014; Timoshevskiy et al. 2019), and it has therefore been suggested that eliminating repetitive DNA might be an important force behind the evolution of this system. In zebra finches, however, the GRC does not seem to have a higher repeat content than the core chromosomes (Kinsella et al. 2019). It will be interesting to determine whether Dipterans with GRCs are like zebra finches in this regard, and whether the amount of recombination on GRCs affects repetitive DNA content on these chromosomes.

Additionally, genomic characterization of germline-restricted DNA from additional lineages will allow us to better understand how this phenomenon evolves. It seems clear that over time, germline-restricted DNA is enriched in genes that function in germline development and reproduction, but it is currently unclear how this system of germline/soma differentiation initially evolves, and whether the same catalysts lead to its evolution in different lineages. As such, understanding how GRCs evolved in Diptera is important, as this phenomenon has evolved at least twice in this order and we currently know little about the origins of GRCs as opposed to lineages with chromatin diminution.

## Outlook and Future Directions

We know a lot about GRCs in Diptera from cytological studies, but very little about what genes they encode, if these genes are expressed and what role they might play. Building upon this extensive cytological knowledge, genomic and functional studies should help remedy this. Dipteran GRCs are particularly interesting for a number of reasons. They have evolved independently several times in relatively closely related taxa, and some features of GRCs, for example, the manner in which GRC elimination occurs from somatic cells, are remarkably similar in Chironomidae, Sciaridae, and Cecidomyiidae. On the other hand, differences between the three Dipteran lineages, for example, in transmission patterns of GRCs, allow for comparisons of the genetic underpinning behind differences. Within-lineage variability in presence and number of GRCs also provides a powerful system for comparative work. Further research on these chromosomes will facilitate broader comparisons with other animal lineages with germline-restricted DNA, and particularly with other lineages with GRCs, such as passerine birds.

There are many remaining questions about GRC evolution in Dipterans which future studies can help resolve. A few of these questions are as follows:

1. What is the origin of GRCs in Chironomidae, Sciaridae, and Cecidomyiidae? Did GRCs in Chironomidae and Cecidomyiidae arise from polyploidization of core chromosomes and do the GRCs in Cecidomyiidae and Sciaridae have a common origin?
2. What is the function of GRCs in the three Dipteran lineages and have similar genes been coopted by GRCs in different lineages (with different origins)?
3. Are the genes on GRCs expressed (particularly in *B. coprophila* that has been shown to have many protein-coding genes on their GRCs) and at what life stage?
4. Do the GRCs have a role in sex determination?
5. How does sex-biased transmission of GRCs affect their evolution?

In order to answer these questions, genomic analyses targeting germ tissue (or early embryonic tissue before GRC elimination) from a wide variety of Dipteran species with GRCs, especially in lineages that have not yet been sampled, is needed. This would allow for detailed phylogenetic analyses of both the GRC and the core genome genes across the different clades. Furthermore, population genomic analyses of GRC-linked polymorphisms could help uncover the patterns of selection acting on these chromosomes. RNAseq and proteomic data from germ tissue is also essential to help resolve which genes on GRCs are functionally important. Flies provide a promising opportunity for experimental validation of GRC function. Many species can be easily kept in the lab and previous work has shown that, unlike in birds, for example, GRC number and presence can be manipulated in the lab. Also, gene editing techniques are well developed for many Dipteran species and could aid more fine-scale studies of individual GRC genes. All of this together makes flies an ideal system to study the function and evolution of germline-restricted DNA. By doing so, this work has the potential to provide key insights into many fundamental aspects of evolutionary genetics and chromosome evolution.

## Glossary

**Germline-restricted chromosomes (GRCs):** chromosomes that are eliminated from somatic cells early in development, so they are found only in germ cells in adults of species that carry them.

**Core chromosomes:** the nongermline-restricted part of the genome including sex chromosomes and autosomes. Please note that core chromosomes occur both in the soma and in the germline.

**Programmed DNA elimination:** a process in which certain regions of the genome are eliminated from some cells in a regulated manner early in development.

**Chromatin diminution:** a type of programmed DNA elimination in which portions of chromosomes are eliminated from somatic cells early in development, leading to portions of the genome being restricted to the germline.

**Chromosome elimination:** a type of programmed DNA elimination in which entire chromosomes are eliminated from somatic cells early in development, leading to certain chromosomes being restricted to the germline.

**Paternal genome elimination:** a non-Mendelian system of reproduction where meiosis is unconventional in males such that they transmit exclusively maternally inherited chromosomes to offspring. Sciaridae and Cecidomyiidae exhibit this form of reproduction.

**Monogeny:** species in which females only produce offspring of one sex (i.e., females are either male producers or female producers). As opposed to **digeny** where females produce offspring of both sexes. Most sexual animals are digenic.
